# Novel SNARE Complex Polymorphisms Associated with Multiple Sclerosis: Signs of Synaptopathy in Multiple Sclerosis

**DOI:** 10.4274/balkanmedj.galenos.2018.2017.1034

**Published:** 2019-05-10

**Authors:** Osman Özgür Yalın, Tuba Gökdoğan Edgünlü, Sevim Karakaş Çelik, Ufuk Emre, Taşkın Güneş, Yüksel Erdal, Aysun Eroğlu Ünal

**Affiliations:** 1 Clinic of Neurology, İstanbul Training and Research Hospital, İstanbul, Turkey; 2 Department of Medical Biology, Muğla Sıtkı Koçman University School of Medicine, Muğla, Turkey; 3 Department of Molecular Biology and Genetic, Zonguldak Bülent Ecevit University Faculty of Science, Zonguldak, Turkey; 4 Clinic of Neurology, İstanbul Bahçelievler State Hospital, İstanbul, Turkey; 5 Department of Neurology, Tekirdağ Namık Kemal University School of Medicine, İstanbul, Turkey

**Keywords:** Multiple sclerosis, polymorphism, SNARE proteins

## Abstract

**Background:**

It is well known that axonal degeneration plays a role in disability in patients with multiple sclerosis, and synaptopathy has recently become an important issue.

**Aims:**

To investigate the possible roles of selected synaptic and presynaptic membrane protein genetic polymorphisms (VAMP2, SNAP-25, synaptotagmin, and syntaxin 1A) in patients with multiple sclerosis.

**Study Design:**

Case-control study.

**Methods:**

A total of 123 patients with multiple sclerosis and 192 healthy controls were included. The functional polymorphisms of specific SNARE complex proteins (VAMP2, synaptotagmin XI, syntaxin 1A, and SNAP-25) were analyzed by polymerase chain reaction.

**Results:**

Significant differences were detected in the genotype and allele distribution of 26-bp Ins/Del polymorphisms of VAMP2 between patients with multiple sclerosis and control subjects; Del/Del genotype and Del allele of VAMP2 were more frequent in patients with multiple sclerosis (p=0.011 and p=0.004, respectively). Similarly, Ddel polymorphism of SNAP-25 gene C/C genotype (p=0.059), syntaxin 1A T/C and C/C genotypes (p=0.005), and synaptotagmin XI gene C allele (p=0.001) were observed more frequently in patients with multiple sclerosis. CC, syntaxin rs1569061 1A gene for 33-bp promoter region TC haplotypes, and synaptotagmin XI gene were found to be associated with an increased risk for multiple sclerosis (p=0.012). Similarly, GC haplotype for rs3746544 of SNAP-25 gene and rs1051312 of SNAP-25 gene were associated with an increased risk for multiple sclerosis (p=0.022).

**Conclusion:**

Genetic polymorphisms of SNARE complex proteins, which have critical roles in synaptic structure and communication, may play a role in the development of multiple sclerosis.

Multiple sclerosis (MS) is an autoimmune, inflammatory disorder related to neuronal structure, and it is one of the prominent causes of young and middle-aged adult disability in the community. Several factors have been proposed for the development of MS, including genetic and environmental. Large population-based studies have shown that a high incidence of family history is possibly related to genetic factors (1). Specific genes associated with MS include the human leukocyte antigen system locus on chromosome 6, which serves as a major histocompatibility complex. The most consistent associations with MS have been found with DR15 and DQ6 alleles. Genome-wide association studies have also demonstrated several other susceptible genes besides the human leukocyte antigen loci (2,3).

Clinical presentation of MS can include various typical syndromes, including optic neuritis, pyramidal signs, and brainstem syndromes. However, consensus exists about the various clinical courses of the disease and the different types of MS (relapsing and progressive forms) based on clinical and pathological features.

MS is traditionally characterized based on chronic inflammatory demyelinating lesions (4); however, recent studies have reported that axonal degeneration may be prominent in the beginning of the inflammation period of the disease (5,6). Axonal damage could be caused due to inflammation, vulnerability of demyelinated axons, or toxic effects of various complex biochemical reactions (7). Accumulating evidence indicates that axonal degeneration occurs not only as a consequence of inflammatory demyelination, but it can also progress independently (5). Furthermore, axonal degeneration is assumed as one of the major determinants of permanent neurological impairment (8,9), and more recent studies have been focusing on the neurodegenerative component of the disease, which is believed to cause disability.

Experimental autoimmune encephalomyelitis is a useful model for the development of new therapies for MS (10). The levels of synapsins and syntaxins are decreased in experimental autoimmune encephalomyelitis. Synapsins and syntaxins are vesicle proteins that exhibit principal roles in exocytosis and in presynaptic terminals in the clinical symptoms of experimental autoimmune encephalomyelitis (11).

There is accumulated evidence suggesting the role of synaptopathy in MS. The term synaptopathy defines the alterations of the synaptic structure and function that have been associated in various neurological diseases, including epilepsy, autism, Alzheimer’s disease, and recently MS (12). Inflammatory-dependent synaptopathy is of particular interest because it is potentially reversible and may represent a novel therapeutic target for MS (12). Synapsins are neuronal phosphorylated proteins and play significant roles in vesicular trafficking in the synaptic region and are particularly related to cytoplasmic vesicle membranes (13). Syntaxin 1A protein is located in the presynaptic region and forms the SNARE complex with SNAP-25 and VAMP2; the SNARE complex is critical in neurotransmission (14). SNAP-25 plays a role in vesicle docking and fusion by mediating neurotransmitter secretion (15). SNAP-25 is present in the neurons and highly located in synaptic plasticity locations (16). Vesicle-associated SNARE complex components induce membrane fusion.

Recent studies have been focusing on factors that determine disability (6,8,9). Synaptopathy, neuroplasticity, axonal degeneration, and accumulated oxidative stressors are some of the proposed mechanisms. Therefore, we examined the synaptic vesicle protein (VAMP2), syntaxin 1A, and SNAP-25 genetic variants that are related to synaptogenesis and neuroplasticity in patients with MS.

## MATERIALS AND METHODS

All patients voluntarily participated in the study, and consent was obtained from all participants. The Ethical Committee of the İstanbul Training and Research Hospital, Fatih, İstanbul approved this nonprofit study. The study was conducted between February 2014 and November 2015 in the neurology outpatient and inpatient clinics at the İstanbul Training and Research Hospital. Informed consents obtained from all participants. A total of 123 subjects with MS aged >18 years and 192 age-matched and healthy controls were included in this study. All the patients were evaluated and underwent a detailed neurologic examination by an experienced neurology specialist. After the final evaluation, the diagnosis of MS was based to the 2010 McDonald criteria (17). Finally, 87 patients with relapsing-remitting multiple sclerosis, 32 patients with secondary progressive multiple sclerosis, 4 patients with primary progressive multiple sclerosis, and 123 patients with MS were enrolled in this study. Those with clinically isolated syndrome, neuromyelitis optica, other demyelinating diseases, and doubtful cases were excluded.

First, we performed a literature search for identifying the associations of syntaxin 1A (intron 7, rs1569061), SNAP-25 (MnlI, rs3746544, and DdeI, rs1051312), VAMP2, and synaptotagmin XI gene polymorphisms in the MS population (Table 1) (14,15,16).

### Genotype analysis

Blood samples were collected from patients and healthy volunteers in tubes containing ethylenediaminetetraacetic acid. Samples were stored at -20 °C until DNA isolation. DNA was isolated from whole blood using Pure Link Genomic DNA Purification Kit (Thermo Massachusetts, USA). Polymerase chain reaction was performed using 2 mM dNTPs (Thermo Scientific R0242), 10 pmol of each primer, 1.5 mM MgCl_2_, and 1×polymerase chain reaction buffer containing (NH_4_)2SO_4_ and 2 U Taq DNA polymerase (Thermo Scientific 0402 Massachusetts, USA). Amplification was performed on an automated thermal cycler (Techne Flexigene, Cambridge, UK). A 100-bp DNA ladder (Thermo Scientific) was used as standard size for each gel lane. The gel was visualized under a UV visualizing system (Vilber Eberhardzell, Germany).

### Statistical analysis

Power analysis was performed considering the type I error rate as 0.05 for VAMP2 variable with 0.17 effect size, and the power was calculated as 0.775. Hardy–Weinberg equilibrium analysis was performed for patients and the control group, and the results were verified using the chi-square test and by estimating the expected genotypic frequencies based on the development of the square of the binomial. Baseline characteristics of the patients and control subjects were compared using t tests for continuous variables, and the results were presented as mean ± standard deviations, and χ2 tests were used for categorical variables. Allelic and genotypic distributions between the study groups were compared using likelihood-ratio chi-square test or Fisher’s exact test. Linkage disequilibrium was tested, and haplotype analysis was performed to investigate the effects of the “linked” genes. Frequencies <0.03 were neglected. Statistical analysis was performed using SPSS 11.5 for Windows.

## RESULTS

This study included 123 patients with MS aged 18-65 years and 192 healthy controls. The patient group comprised 87 patients with relapsing-remitting multiple sclerosis, 32 patients with secondary progressive multiple sclerosis, and 4 subjects diagnosed with primary progressive multiple sclerosis. The mean age of the patients was 44.8±10.0 years, and that of the control group was 42.9±11.3 years. There was no significant difference in age and gender (p=0.268) between the groups. The demographic characteristics of the study subjects are presented in Table 2.

DNA samples collected from all participants were evaluated. We analyzed the alleles and genotypes of 26-bp Ins/Del polymorphisms of the VAMP2 gene, intron 7 rs1569061 polymorphism of the syntaxin 1A gene, MnII (rs3746544) and Ddel (rs1051312) polymorphisms of the SNAP-25 gene, and 33-bp repeats in promoter regions of the synaptotagmin XI gene.

Significant differences were observed in the genotype distribution of the 26-bp Del/Del polymorphisms of the VAMP2 and Del/Del genotypes, which were 3.194 (range 1.463-6.974) times more common in patients with MS (p=0.012). No difference was observed for the SNAP-25 gene MnII (rs3746544) polymorphism; however, Ddel (rs1051312) polymorphism C/C genotype was more frequently found in patients with MS [odds ratio (OR) (95% confidence interval (CI) 2.137 (1.075-4.248), p=0.059]. Polymorphisms of intron 7 rs1569061 syntaxin 1A gene T/C and C/C genotypes were observed significantly more frequently in the MS group. This difference was also observed in patients with relapsing-remitting multiple sclerosis [OR (95% CI) 2.127 (1.225-3.693) and 2.544 (1.366-4.738), respectively, p=0.05]. The results of the genotypes of VAMP2, SNAP-25, and syntaxin 1A gene variants are presented in Table 3.

We investigated the alleles of VAMP2, SNAP-25, syntaxin 1A, and synaptotagmin XI gene polymorphisms. Del allele of VAMP2 gene was observed 1.688 (range 1.183-2.407) times more frequently in patients with MS (p=0.004); similarly, the C allele of synaptotagmin XI gene was observed more frequently in patients with MS [OR (95% CI) 1.711 (1.238-2.363), p=0.001]. Allelic distributions of other genes were similar for both groups. The results are presented in Table 4.

Finally, haplotype analysis revealed significant differences between the study groups. We observed that TC and CC haplotypes for rs1569061 syntaxin 1A gene and the 33-bp promoter region of the synaptotagmin XI gene could be risk factors for the development of MS [OR (95% CI) 1.686 (1.049-2.710) and 1.588 (1.041-2.420), p=0.012, respectively]. Similarly, the GC haplotype of the SNAP-25 Mnll gene and the SNAP-25 Ddel gene was associated with a 1.948-fold [OR (95% CI) 1.948 (1.251-3.033), p=0.022) increased risk of MS development. Results of the haplotype analysis are presented in Table 5.

The data of patients with secondary progressive multiple sclerosis and primary progressive multiple sclerosis were not analyzed separately because of the small number of patients.

## DISCUSSION

We evaluated the polymorphisms of the SNARE complex proteins (VAMP2, SNAP-25, syntaxin 1A, and synaptotagmin XI) in patients with MS. To our knowledge, this is the first study to evaluate the associations between the SNARE complex genetic polymorphisms and MS. We found associations between MS and VAMP2 Del/Del, SNAP-25 Ddel C/C, and syntaxin1A T/C and C/C genotypes. VAMP2 gene Ddel allele and C allele of synaptotagmin XI were also associated with an increased risk of developing MS. Moreover, syntaxin1A/synaptotagmin XI genes, TC and CC haplotypes, and SNAP-25 Mnll/SNAP-25 Ddel GC haplotype were found to be associated with an increased risk of MS development.

It has recently been proposed that not only myelin but also synapses are involved in the early phases of MS development (18,19). Initial autopsy studies of patients with relapsing-remitting multiple sclerosis, primary progressive multiple sclerosis, and secondary progressive multiple sclerosis have reported decreased levels of synaptic proteins, including synaptotagmin and synaptophysin. Synaptophysin is a presynaptic vesicle protein that plays a role in synaptic vesicle release (20,21).

Evidence of early synaptopathy in MS has been documented in experimental and autopsy studies (12,21,22). Earlier studies investigating synapsin III genetic polymorphisms in MS have reported conflicting results (23,24,25). SNARE proteins exist in all parts of the brain tissue. There is accumulated evidence showing that SNARE proteins are widely present in the brain. SNARE complex proteins play a crucial role in providing and sustaining healthy synaptic structure and functions. Genetic polymorphisms of associated proteins have been found to be associated with several central nervous system disorders (26,27,28,29). Synapses are morphologically dynamic structures and dysfunctions can be repaired and new synapses can be formed. This dynamic structure of synapses could provide novel treatment strategies (12). The SNARE-related genes may have a role in structural expression or may contribute to neurological disorders. As a part of the SNARE complex, synaptotagmins are extensively found in the brain and have important roles in membrane trafficking and synaptic plasticity. Synaptotagmin polymorphisms have been well studied in the etiology of epilepsy (29).

Synapsins are composed of a group of specific neuroproteins. Synapsins are responsible for trafficking of synaptic vesicles from presynaptic terminals, particularly related to the cytoplasmic surface (30). They play a crucial role in synaptogenesis and early axonal development. Although SNAP-I and -II are believed to be associated with neuronal development, SNAP-III was recently discovered and has unique functions in neurotransmitter regulation in mature neurons (31). The role of SNAP-III polymorphisms has been investigated in schizophrenia, Alzheimer’s disease, and MS (31,32,33,34). Liquori et al. (31) reported an inverse association between SNAP-III promoter gene polymorphism and MS. SNAP-25 is another SNARE complex protein that has been investigated in psychiatric and neurological diseases. SNAP-25 is believed to play a role in regulating synaptic homeostasis (35). In particular, syntaxin 1A has a role in the synaptic exocytosis process and neuronal plasticity (36). Although the roles of presynaptic and synaptic membrane proteins have been well investigated, their precise roles in the healthy brain and in neurological disorders still remain unclear.

This study has certain limitations; it is not population-based and may not represent the wider population. The sample size of the study group was relatively small to assess the secondary progressive multiple sclerosis and primary progressive multiple sclerosis genetic associations and the correlations with disability scores. We are planning to investigate a broader series of relationships of genetic polymorphisms in our future studies.This study has demonstrated novel genetic associations in MS related to synaptopathy. We believe that future functional and clinical studies on the gene expressions of SNARE complex proteins are needed. Our findings should be confirmed by further studies in different populations.

## Figures and Tables

**Table 1:**
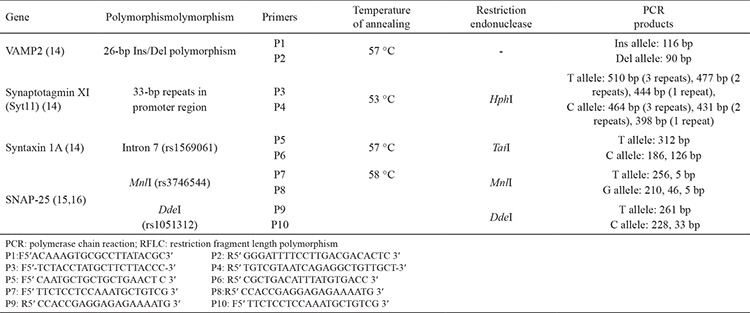
The PCR-RFLP evaluation method for VAMP2, synaptotagmin XI, syntaxin 1A, and SNAP-25 genes

**Table 2:**
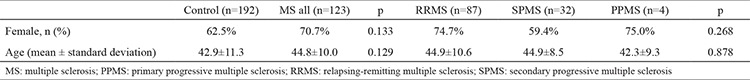
Demographic and clinical features of the study population

**Table 3:**
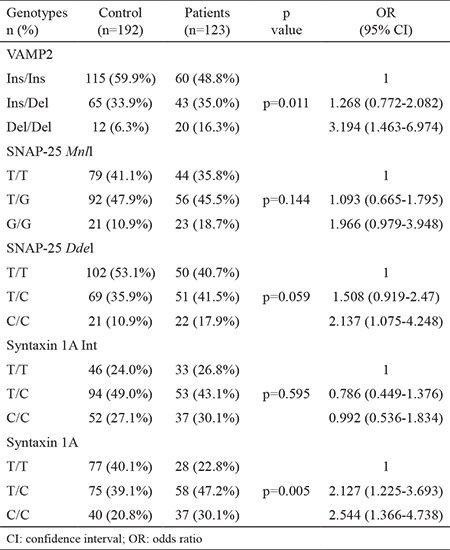
Genotypes of VAMP2, SNAP-25, Syntaxin 1A genes

**Table 4:**
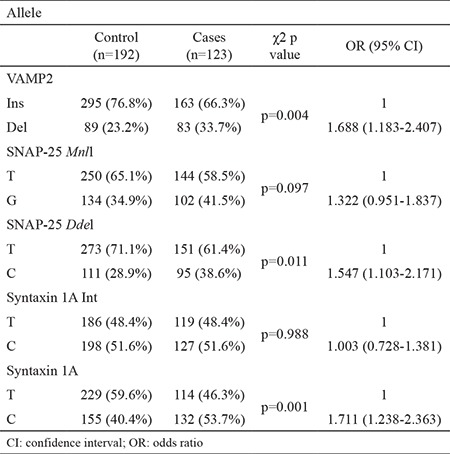
Alleles of VAMP2, SNAP-25, and syntaxin 1A genes

**Table 5:**
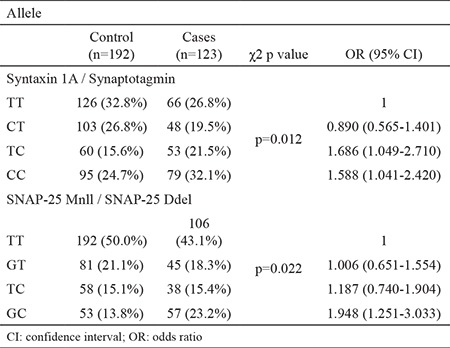
Haplotype distributions of Syntaxin 1A and SNAP-25 genes
